# Pulmonary Emphysema Regional Distribution and Extent Assessed by Chest Computed Tomography Is Associated With Pulmonary Function Impairment in Patients With COPD

**DOI:** 10.3389/fmed.2021.705184

**Published:** 2021-09-23

**Authors:** Plácido Gomes, Hélder Novais e Bastos, André Carvalho, André Lobo, Alan Guimarães, Rosana Souza Rodrigues, Walter Araujo Zin, Alysson Roncally S. Carvalho

**Affiliations:** ^1^Faculty of Medicine, Universidade do Porto, Porto, Portugal; ^2^Serviço de Pneumologia, Centro Hospitalar de São João EPE, Porto, Portugal; ^3^Instituto de Investigação e Inovação em Saúde, Universidade do Porto, Porto, Portugal; ^4^Instituto de Biologia Molecular e Celular, Universidade do Porto, Porto, Portugal; ^5^Serviço de Radiologia, Centro Hospitalar de São João EPE, Porto, Portugal; ^6^Centro Hospitalar Vila Nova de Gaia/Espinho, Porto, Portugal; ^7^Laboratory of Pulmonary Engineering, Biomedical Engineering Program, Alberto Luiz Coimbra Institute of Post-Graduation and Research in Engineering, Universidade Federal do Rio de Janeiro, Rio de Janeiro, Brazil; ^8^Department of Radiology, Universidade Federal Do Rio de Janeiro, Rio de Janeiro, Brazil; ^9^IDOR–D'Or Institute for Research and Education, Rio de Janeiro, Brazil; ^10^Laboratory of Respiration Physiology, Carlos Chagas Filho Institute of Biophysics, Universidade Federal do Rio de Janeiro, Rio de Janeiro, Brazil; ^11^Cardiovascular R&D Center, Department of Surgery and Physiology, Faculty of Medicine of the University of Porto, Porto, Portugal

**Keywords:** chronic obstructive pulmonary disease, pulmonary emphysema, computed tomography, CT-estimated emphysema, quantitative chest CT analysis

## Abstract

**Objective:** This study aimed to evaluate how emphysema extent and its regional distribution quantified by chest CT are associated with clinical and functional severity in patients with chronic obstructive pulmonary disease (COPD).

**Methods/Design:** Patients with a post-bronchodilator forced expiratory volume in one second (FEV_1_)/forced vital capacity (FVC) < 0.70, without any other obstructive airway disease, who presented radiological evidence of emphysema on visual CT inspection were retrospectively enrolled. A *Qua*ntitative *L*ung *I*maging (QUALI) system automatically quantified the volume of pulmonary emphysema and adjusted this volume to the measured (EmphCT_LV_) or predicted total lung volume (TLV) (EmphP_LV_) and assessed its regional distribution based on an artificial neural network (ANN) trained for this purpose. Additionally, the percentage of lung volume occupied by low-attenuation areas (LAA) was computed by dividing the total volume of regions with attenuation lower or equal to −950 Hounsfield units (HU) by the predicted [LAA (%P_LV_)] or measured CT lung volume [LAA (%CT_LV_)]. The LAA was then compared with the QUALI emphysema estimations. The association between emphysema extension and its regional distribution with pulmonary function impairment was then assessed.

**Results:** In this study, 86 patients fulfilled the inclusion criteria. Both EmphCT_LV_ and EmphP_LV_ were significantly lower than the LAA indices independently of emphysema severity. CT-derived TLV significantly increased with emphysema severity (from 6,143 ± 1,295 up to 7,659 ± 1,264 ml from mild to very severe emphysema, *p* < 0.005) and thus, both EmphCT_LV_ and LAA significantly underestimated emphysema extent when compared with those values adjusted to the predicted lung volume. All CT-derived emphysema indices presented moderate to strong correlations with residual volume (RV) (with correlations ranging from 0.61 to 0.66), total lung capacity (TLC) (from 0.51 to 0.59), and FEV_1_ (~0.6) and diffusing capacity for carbon monoxide DL_CO_ (~0.6). The values of FEV_1_ and DL_CO_ were significantly lower, and RV (*p* < 0.001) and TLC (*p* < 0.001) were significantly higher with the increasing emphysema extent and when emphysematous areas homogeneously affected the lungs.

**Conclusions:** Emphysema volume must be referred to the predicted and not to the measured lung volume when assessing the CT-derived emphysema extension. Pulmonary function impairment was greater in patients with higher emphysema volumes and with a more homogeneous emphysema distribution. Further studies are still necessary to assess the significance of CTpLV in the clinical and research fields.

## Introduction

Chronic obstructive pulmonary disease (COPD) presents important morbidity and mortality worldwide with a prevalence as high as 14.2% in some areas of Portugal (1–3). COPD is characterized by persistent respiratory symptoms and airflow limitation caused by the pathophysiological changes ranging from chronic inflammation and narrowing of peripheral airways to emphysema (4–7).

The patients with predominant emphysema phenotype exhibit a more severe lung function impairment, such as decreased gas exchange efficiency, lower forced expiratory volume in one second (FEV_1_), and FEV_1_/forced vital capacity (FVC) ratio (8–10).

The correlation between the CT-derived emphysema indices and lung function impairment has been well-established (11, 12). However, only recent advances allowed the quantification of emphysema distribution by CT and its association with different degrees of clinical severity (4, 13–15). None of these reports considered that hyperinflation is a major characteristic of emphysematous patients, which would increase total lung volume (TLV), possibly skewing the measurement of emphysema extent (16). The adjustment of emphysema volume to the predicted TLV might be of potential interest when one wishes to assess disease progression, since the increase in TLV may lead to a progressive underestimation of the extent of emphysema (13, 17, 18).

In the present study, we employed a *QUA*ntitative *L*ung *I*maging (QUALI) system as a computer-aided diagnosis (CAD) system that applies a previously proposed artificial neural network (ANN) to automatically classify and locate the lung patterns commonly used in clinical practice: emphysema, normal parenchyma, ground-glass opacity (GGO), crazy paving and linear opacities (CP/LO), and consolidation from the chest CT images (19). Additionally, low attenuation areas (LAA) were also calculated as the percentage of lung volume occupied by voxels with attenuation of −950 Hounsfield Units (HU) or less (6, 20, 21).

Thereafter, we aimed to evaluate whether the extension of emphysema quantified by several indices derived from CT-scan images and its regional distribution are associated with the clinical and functional features in emphysematous patients.

## Methods

### Study Design and Study Subjects

**Figure 1:**
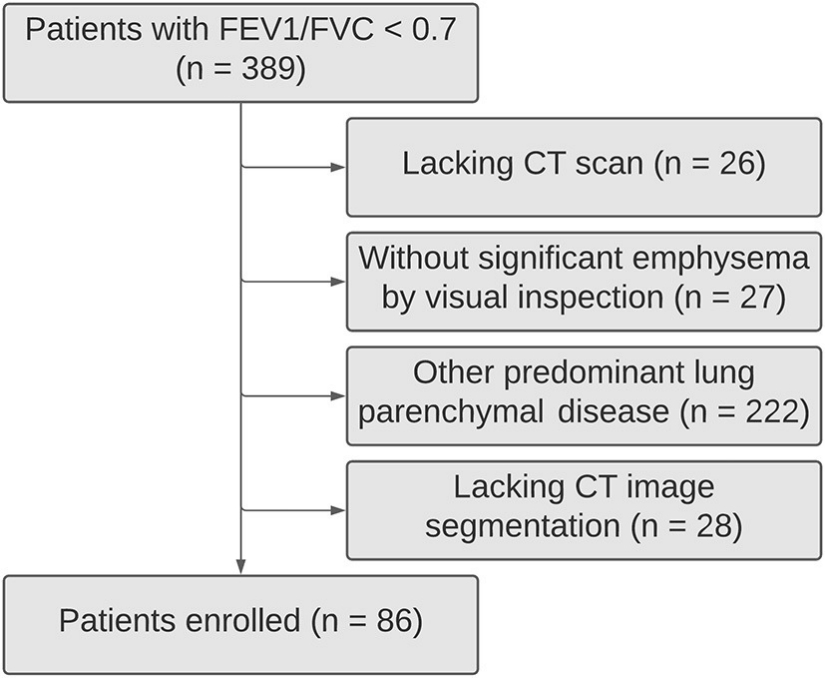
Patient flowchart.

Patients from the Department of Pulmonology of Centro Hospitalar e Universitário de São João, Porto, Portugal, with a post-bronchodilator FEV_1_/FVC <0.70 were retrospectively selected. The patients with <18 years old and a history of asthma, bronchiectasis, tuberculosis sequelae, cystic fibrosis, lung fibrosis, asthma/COPD overlap syndrome, thoracic surgery, or other confounding diseases were excluded (Figure 1).

### Chest CT Acquisition and Image Processing

The CT scans were done with either a 32-channel SOMATOM go. Up or a 16-channel SOMATOM go. Now (Siemens Healthineers, Germany). The acquisitions were gathered in the supine position with 120 kV and 120–300 mA, slice thickness ranging from 3 to 5 mm without superposition, and a 512 × 512, 768 × 768, or 1,024 × 1,024 voxels matrix.

First, we visually inspected the thoracic CT scans of the selected patients in images reconstructed with a soft tissue (standard) convolutional kernel. Only patients showing evidence of emphysema, and tested for pulmonary function [spirometry, body plethysmography and diffusing capacity for carbon monoxide (DL_CO_)] within a year prior to the CT-scan were included.

**Figure 2:**
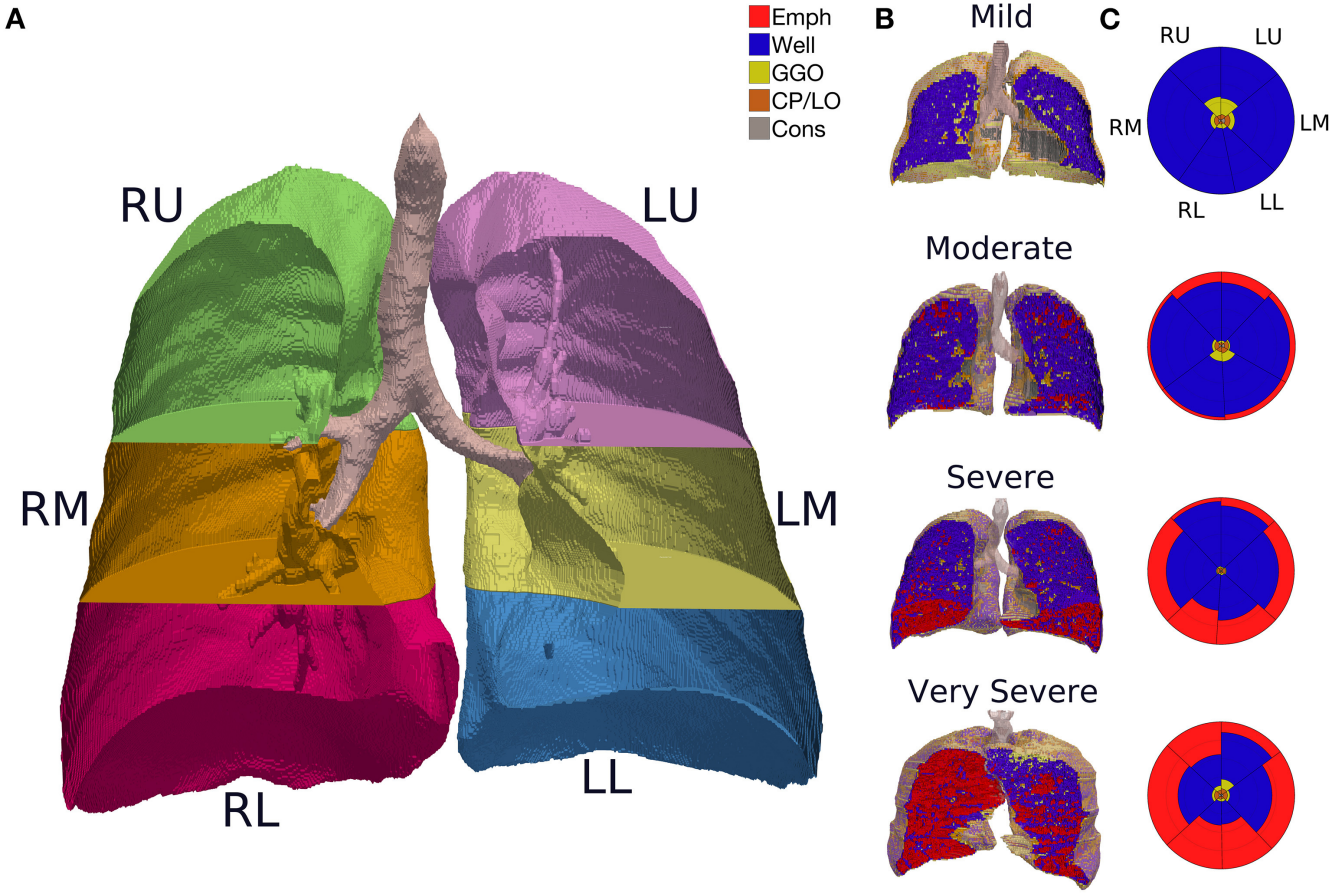
**(A)** CT image segmentation in a representative subject. R, right lung; L, left lung; U, upper lung; M, middle lung; L, lower lung. **(B)** QUALI system classification depicted in a 3D scan renderization from representative patients with mild, moderate, severe, or very severe emphysema cases. Mild: FEV_1_ ≥ 80%; moderate 50–79%; severe 30–49%; very severe <30% of the predicted value. Red represents emphysema, yellow are GGO, orange means CP/LO, and gray represent consolidation. **(C)** Summary glyphs corresponding to the underlying 3D scan data. In each glyph, the first letter (R/L) indicates the right and left lung, the second letter (U/M/L) denotes, respectively, upper, middle, and lower lung zones.

Thereafter, the CT images series reconstructed with a sharp (bone) convolution kernel were selected and lung parenchyma and airways were automatically segmented by applying the Region Growing algorithm using the module Chest Imaging Platform (CIP) and segmentation tool with the Generate Partial Lung Map Label (3D Slicer version 4.8.1 software) (22). The right and left lungs were vertically divided into three equal segments (Figure 2A). All the images were visually inspected, manually edited, and exported to an in-house developed software (QUALI) written in MATLAB® (MathWorks®, Natick, MA, USA) (19).

After lung segmentation, CT-derived TLV was computed as:


(1) $$\begin{array}{r} \text{C}{\text{T}}_{\text{LV}}\left(\text{ml}\right)=\text{pixel siz}{\text{e}}^{2}\times \text{slice thickness}\\ \times \text{total number of pixels} \end{array}$$

The predicted TLV for a healthy person lying supine in deep inspiration was computed as previously reported:


(2) $$\begin{array}{l} {\text{P}}_{\text{LV}}\left(\text{ml}\right)=4808.1\times \text{height }\left(\text{m}\right)-3602.5 \end{array}$$

for males 800.6 ml should be added to P_LV_ (23).

### Development of the Supervised Neural Network Architecture

Two chest radiologists blinded to patient identification, clinical data, and outcomes independently selected regions of interests (ROIs) from the 228 chest CT-scan images of patients with SARS-CoV-2 and 30 patients with pulmonary emphysema to visually classify the ROIs as emphysema regions (Emph, *n* = 14,162), normal parenchyma (*n* = 31,785), ground-glass opacities (GGO, *n* = 2,369), crazy paving/linear opacities (CP/LO, *n* = 7,337), and consolidation (*n* = 16,053), totaling 71,706 ROIs.

This ROI dataset comprises images acquired with several CT equipment, such as a 64-channel multislice (Brilliance 40 scanner, Philips Medical Systems, Cleveland, OH, USA and General Electrics Lightspeed VCT, Chicago, IL, USA), a 128-channel multislice dual-source CT system (Somatom Definition Flash, Siemens, Forchheim, Germany), and a 16-channel multislice (Emotion 16 CT, Siemens, Erlangen, Germany). Moreover, the images were reconstructed with several convolution kernels (B50f, B60f, B70s, C, FC13, FC86, I50f2, L, LUNG, and SOFT), depending on the manufacturer of CT.

The ROIs were then balanced by the lowest number of ROIs (2,369 ROIs). Thus, the percentiles 2.5, 25, 50, 75, and 97.5% derived from the density histograms of 11,845 ROIs were used for ANN training (8,291 ROIs), validation (1,777 ROIs), and test (1,777 ROIs). Each ROI consisted of a circle with a fixed radius of 4 mm with a spanning area of about 30 voxels in each CT section.

The whole lung CT scans were processed by the proposed QUALI system (19), resulting in a 3D scan renderization that includes all the classes (Figure 2B) and a summary glyph (Figure 2C) to the underlying 3D scan data.

### Determination of Emphysema Extent

From the ANN-based classification procedure, the extent of emphysema was assessed as the cumulative sum of the QUALI-referred emphysema areas adjusted to CT_LV_ (EmphyCT_LV_) or P_LV_ (EmphP_LV_).

Additionally, the low attenuation areas (LAA) were calculated accounting for the volume occupied by voxels with attenuation of −950 HU or less (21). LAA index was then adjusted to CT_LV_ [LAA (%CT_LV_)] or P_LV_ [LAA (%P_LV_)].

For the assessment of the regional distribution of emphysema, we verified that at least 5% of each third of the lung was classified as emphysematous regions (24).

### Clinical and Pulmonary Function Assessment

The medical records were reviewed retrospectively regarding demographic and anthropometric data (sex, age, height, weight, and body mass index [BMI]), smoking status (current smoker, former smoker, or non-smoker), inhaled corticosteroid therapy, dyspnea severity as determined by the modified Medical Research Council (mMRC) scale (25), and number of exacerbations in the year before enrolment (26). The patients with pulmonary function tests (PFTs) taken within a year prior to enrollment and at least 6 months without any clinical exacerbation were accepted and their air flow, lung volumes, and DL_CO_ were recorded, in accordance with the international guidelines (27, 28).

### Statistical Analysis

The normality of the data (Kolmogorov–Smirnov test with Lilliefors' correction) and the homogeneity of variances (Levene's median test) were tested. Since all the variables had normal distribution and homogeneous variances, they were expressed as means and SD; categorical variables as absolute values and proportion.

A paired *t*-test was used to compare CT-derived emphysema areas adjusted or not to P_LV_. The Bland–Altman plot was used to evaluate the agreement among EmphP_LV_, EmphCT_LV_, LAA(%P_LV_), and LAA(%CT_LV_).

The correlations among EmphP_LV_, EmphCT_LV_, LAA(%P_LV_), and LAA(%CT_LV_) with pulmonary functional variables were assessed using the Spearman's correlation analysis (very weak, ρ = 0.00–0.19; weak, ρ = 0.20–0.39; moderate, ρ = 0.40–0.59; strong, ρ = 0.60–0.79; very strong, ρ ≥ 0.80).

One-way ANOVA was used to compare the variables among the Global Initiative for Chronic Obstructive Lung Disease (GOLD) severity subgroups. Bonferroni *post-hoc* analysis was applied for multiple comparisons among the subgroups. All the statistical analyses were performed with the SPSS Statistics software package, version 26.0 (IBM Corporation, Armonk, NY, USA).

### Data Management/Ethical Considerations

All data were fairly and lawfully collected by the authors for the specified, explicit, and legitimate purposes and not further processed. The anonymity and confidentiality of collected information and its protection were guaranteed according to the EU Directive 95/46/CE. All data were initially stored on an external disk and later in the cloud with restricted access by user password.

## Results

In this study, 389 subjects had FEV_1_/FVC <0.7 being 86 COPD patients with pulmonary emphysema (Figure 1). Their mean age was 67 ± 11 (SD) years, most of them were men (88.4%), all but two of the patients were current or former smokers. Less than half of the patients were under inhaled corticosteroid therapy (47.7%). Only 26.7% of them had frequent exacerbations (≥2 exacerbations in the last year). Severe symptoms (mMRC dyspnea scale score ≥2) were found in 20.9% of them.

The characteristics of the patients (Table 1) revealed a wide range of airflow limitation according to the GOLD severity classification (29), with a slight predominance of severe airflow limitation: mild, in 14 (16.3%); moderate, in 22 (25.6%); severe, in 36 (41.9%); and very severe, in 14 (16.3%).

**Table 1:** Demographic, clinical, and imaging characteristics of enrolled patients.

	**GOLD severity classification**					
**Clinical features**	**All patients** **(*n* = 86)**	**Mild** **(*n* = 14)**	**Moderate** **(*n* = 22)**	**Severe** **(*n* = 36)**	**Very severe** **(*n* = 14)**	***P*-value**
**Clinical variables**						
Age (years)	67 ± 11	**59.7 ± 13.3**	**69.8 ± 9.1**	**70.1 ± 10.3**	**61.3 ± 8.8**	**0.002**
Male (gender)	76 (88.4%)	12 (85.7%)	17 (77.3%)	34 (97.1%)	13 (92.9%)	
BMI (kg/m^2^)	23.6 ± 4.1	23.3 ± 3	24.7 ± 3.8	23.8 ± 3.8	21.1 ± 5.1	0.07
**Smoking status**						
Previous smokers	53 (61.6%)	6 (42.9%)	17 (77.3%)	22 (62.9%)	7 (50%)	
Current smokers	31 (36%)	8 (57.1%)	4 (18.2%)	12 (34.3%)	7 (50%)	
Exacerbations ≥ 2 previous years	23 (26.7%)	3 (21.4%)	3 (13.6%)	10 (28.6%)	6 (42.9%%)	
Inhaled corticoid therapy	41 (47.7%)	2 (14.3%)	13 (59.1%)	23 (67.5%)	3 (21.4%)	
mMRC dyspnoea score ≥ 2	18 (20.9%)	2 (14.3%)	3 (13.6%)	9 (25.7%)	3 (21.4%)	
**Pulmonary function test variables**						
RV, % of predicted	179.8 ± 59	**124.3 ± 32.5**	**156.6 ± 48.2**	**192.2 ± 40.7**	**268.2 ± 34**	** <0.001**
TLC, % of predicted	120.3 ± 23	**108.4 ± 15.9**	**114.7 ± 24.5**	**124.4 ± 23.4**	**136.3 ± 16.3**	**0.011**
RV/TLC (%)	61.6 ± 22	**46.4 ± 24.2**	**61.7 ± 27.9**	**65.4 ± 17.2**	**71.6 ± 7.4**	**0.021**
FEV_1_, % of predicted	51.3 ± 23	**93.1 ± 27.9**	**61.4 ± 10.3**	**39.4 ± 5.5**	**23.6 ± 3.7**	** <0.001**
DL_CO_, % of predicted	50 ± 22.6	**71.3 ± 15.3**	**62.7 ± 15.4**	**45.2 ± 17.1**	**16.9 ± 7.8**	** <0.001**
DL_CO_/VA, % of predicted	61.6 ± 24	**75.1 ± 14.8**	**73.1 ± 14.6**	**60.1 ± 22.9**	**28 ± 13.2**	** <0.001**
**CT-derived variables**						
TLV (mL)	6680 ± 1398	**6143 ± 1295**	**6200 ± 1391**	**6856 ± 1287**	**7659 ± 1264**	**0.005**
Predicted TLV (mL)	5101 ± 506	5275 ± 511	4976 ± 618	5126 ± 397	5146 ± 458	0.350
LAA (%P_LV_)	17.2 ± 18.4	**5.3 ± 6.8**	**8.5 ± 7.4**	**18.8 ± 18.7**	**38.4 ± 19.8**	** <0.001**
LAA (%CT_LV_)	12 ± 11.6	**4.4 ± 5.4**	**6.4 ± 4.9**	**13 ± 11.4**	**25.4 ± 12.8**	** <0.001**
**QUALI-derived variables**						
EmphP_LV_ (%)	15.7 ± 18^*^	4.6 ± 6.4^*^	6.8 ± 7.0^*^	17.3 ± 18.3^*^	36.5 ± 20.2^*^	** <0.001**
EmphCT_LV_ (%)	10.9 ± 11^‡^	**3.9 ± 5** ^**‡**^	**5.1 ± 4.6** ^**‡**^	**11.9 ± 11.2** ^**‡**^	**24.2 ± 13.1** ^**‡**^	** <0.001**
Emphysema (mL)	804 ± 939	**249 ± 347**	**345 ± 366**	**871 ± 910**	**1918 ± 1104**	** <0.001**
Well-Aerated (mL)	4839 ± 1248	4478 ± 1695	4923 ± 1168	4991 ± 1194	4801 ± 968	0.623
GGO (mL)	487 ± 346	**817 ± 573**	**398 ± 233**	**449 ± 280**	**390 ± 130**	**0.001**
CP/LO (mL)	394 ± 97	433 ± 114	383 ± 86	391 ± 100	383 ± 93	0.436
Consolidation (mL)	156 ± 62	165 ± 49	150 ± 73	153 ± 66	166 ± 50	0.830

*Values are presented as mean ± SD or n (%). GOLD, the Global Initiative for Chronic Obstructive Lung Disease; mMRC, modified Medical Research Council; RV, Residual volume; TLC, Total lung capacity; FEV_1_, Forced expiratory volume measured during the first second; DLCO, Diffusing capacity for carbon monoxide; VA, Alveolar volume; LAA (%P_LV_), Low attenuation areas expressed as a percentage of the predicted lung volume; LAA (%CT_LV_), Low attenuation areas expressed as a percentage of CT measured lung volume; EmphP_LV_ (%), QUALI-derived emphysema volume expressed as a percentage of the predicted lung volume; EmphCT_LV_ (%), QUALI-derived emphysema volume expressed as a percentage of CT measured lung volume. GGO, Ground-glass opacities; CP/LO, Crazy paving/linear opacities.*

* *significantly different from LAA (%P_LV_);*

‡ *significantly different from LAA(%CT_LV_). Bold numbers highlight statistical signficance.

An overall tendency for hyperinflation was clear, with a mean (± SD) RV of 179.8 ± 59% of the predicted value, TLC of 120.3 ± 23% of predicted and a RV/TLC ratio of 61.6 ± 22%. The patients also showed a reduced FEV_1_ of 51.3 ± 23% and DL_CO_ of 50 ± 22.6% of predicted. A significant impairment in pulmonary function was observed from mild to very severe GOLD emphysema severity classification (Table 1).

**Figure 3:**
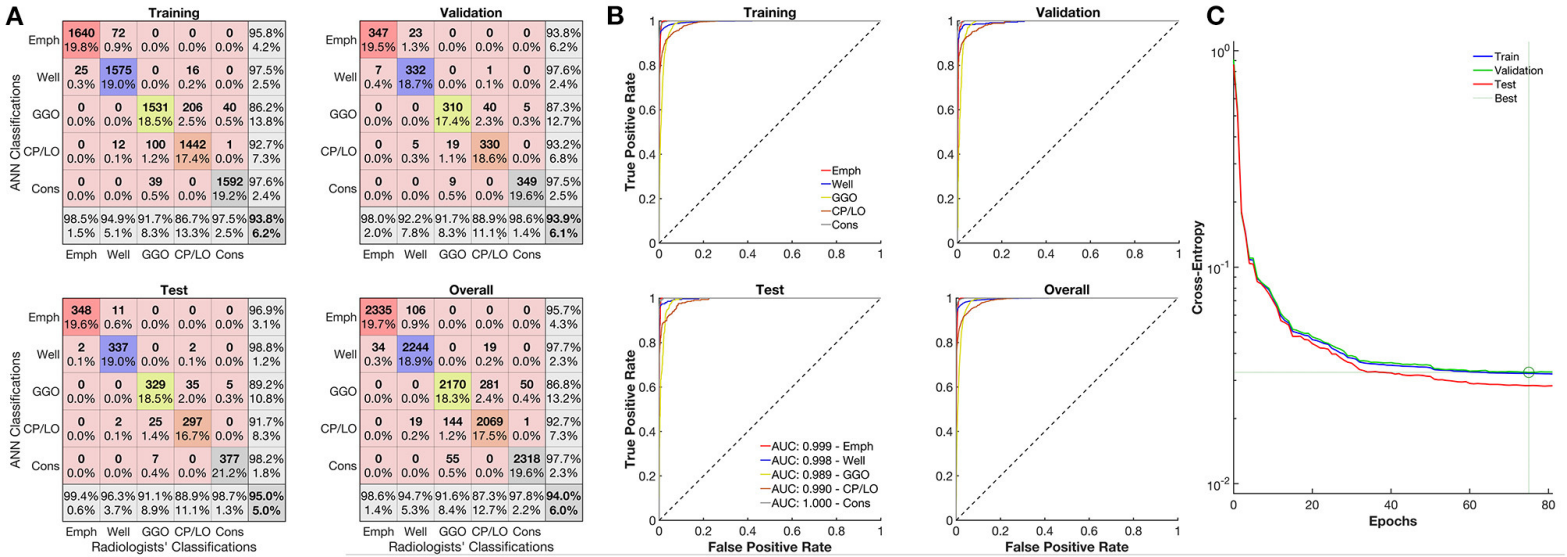
Evaluation of the artificial neural network (ANN) classifier performance. **(A)** Confusion matrix from the comparison between the ANN and classifications of emphysema regions of the radiologists (red line), well-aerated (blue line), GGO (yellow line), CP/LO (orange), and consolidation (gray) in training (first row and column), validation (first row, second column), test (second row, first column), and overall (second row, second column) sets. **(B)** Each respective receiver operator characteristic curve for each class from the ANN classification in training (first row and column), validation (first row, second column), test (second row, first column), and overall (second row, second column) sets. **(C)** Cross-entropy at each epoch in training (blue line), validation (green line), and test (red line) sets. Dotted lines represent the best validation performance based on the minimization of the cross-entropy at epoch 75.

The overall agreement between QUALI and the classifications of radiologists was 95%, being 99.4% for emphysema, 96.3% for well-aerated regions, 91.1% for GGO, 88.9% for CP/LO, and 98.7% for consolidation in the test set (Figure 3A, second row and first column). A receiver operating characteristic (ROC) curve from each radiological pattern is presented in Figure 3B. The ANN classifier performance was higher than 0.99 (0.97–1.0, 95% *CI*) in all classes. The best validation performance occurred at epoch 75 with a minimal entropy of 0.032 (Figure 3C).

**Figure 4:**
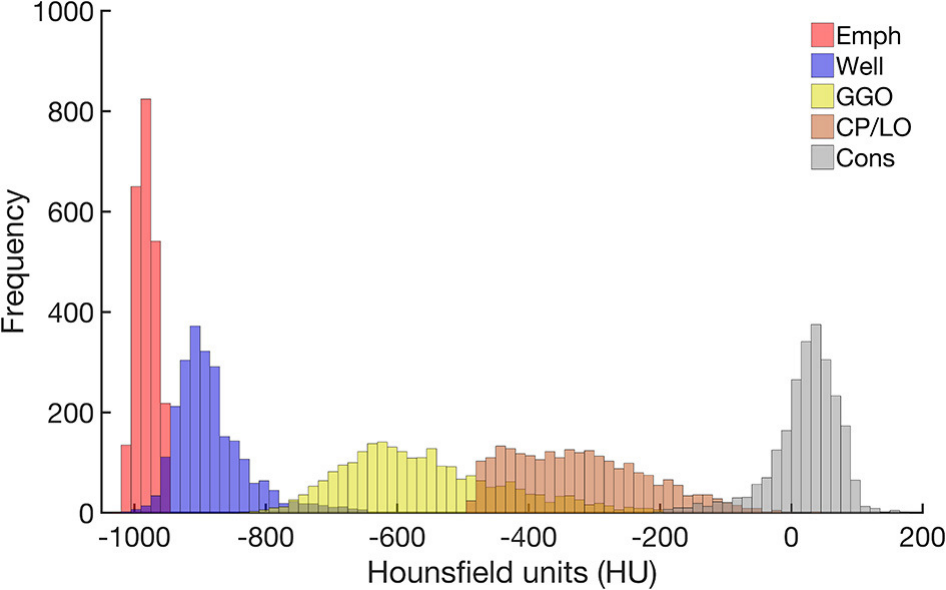
Frequency histograms of the densities expressed in Hounsfield units (HU) from all regions of interest visually assigned as emphysema (red), well-aerated regions (blue), GGO (yellow), CP/LO (orange), and consolidation (light gray). Note the overlapping bins in the highest emphysema densities and the lowest well-aerated densities. This finding putatively contributes to the overestimation of the lower attenuation areas assigned to emphysema and reduces the ANN classifier performance.

The CT density histogram of all ROIs visually assigned as emphysema, well-aerated regions, GGO, CP/LO, and consolidation is depicted in Figure 4. Note that there are several overlapping bins between the densities assigned to normal parenchyma and emphysema, as well as between normal parenchyma and GGO. Additionally, there is an important overlap among GGO, CP/LO, and consolidation, as previously reported (19).

**Figure 5:**
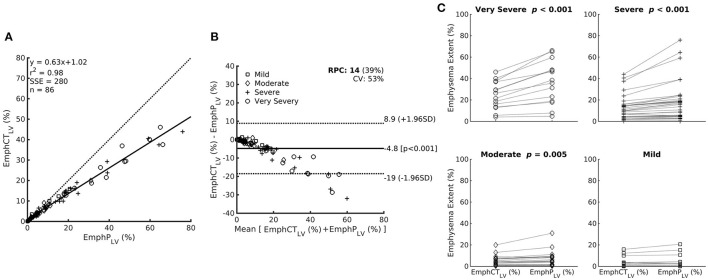
**(A)** Relationship between emphysema estimation by the ANN classifier expressed as a percentage of the CT lung volume (EmphCT_LV_) or as a percentage of the predicted lung volume (EmphP_LV_). Note that EmphCT_LV_ underestimates emphysema extent compared with EmphP_LV_. **(B)** The Bland–Altman bias plot of the relationship between EmphCT_LV_ and EmphP_LV_. **(C)** Paired plot of emphysema extent expressed as EmphCT_LV_ or EmphP_LV_ within the patients with different airflow limitation severity based on post-bronchodilator FEV_1_. Mild: FEV_1_ ≥80%; moderate 50–79%; severe 30–49%; and very severe <30% of the predicted value.

Even though EmphCT_LV_ and EmphP_LV_ were strongly correlated (ρ^2^ = 0.98; ρ = 0.98 and *p* < 0.001), a significant bias of −4.8% (±2 SD ranging from −19 to 8.9%, *p* < 0.001) was observed especially in the subjects with higher emphysema content (EmphCT_LV_ > 20%) (Figures 5A,B). In fact, in all but mild GOLD severity classification, EmphP_LV_ was always significantly higher than EmphCT_LV_ (Figure 5C).

**Figure 6:**
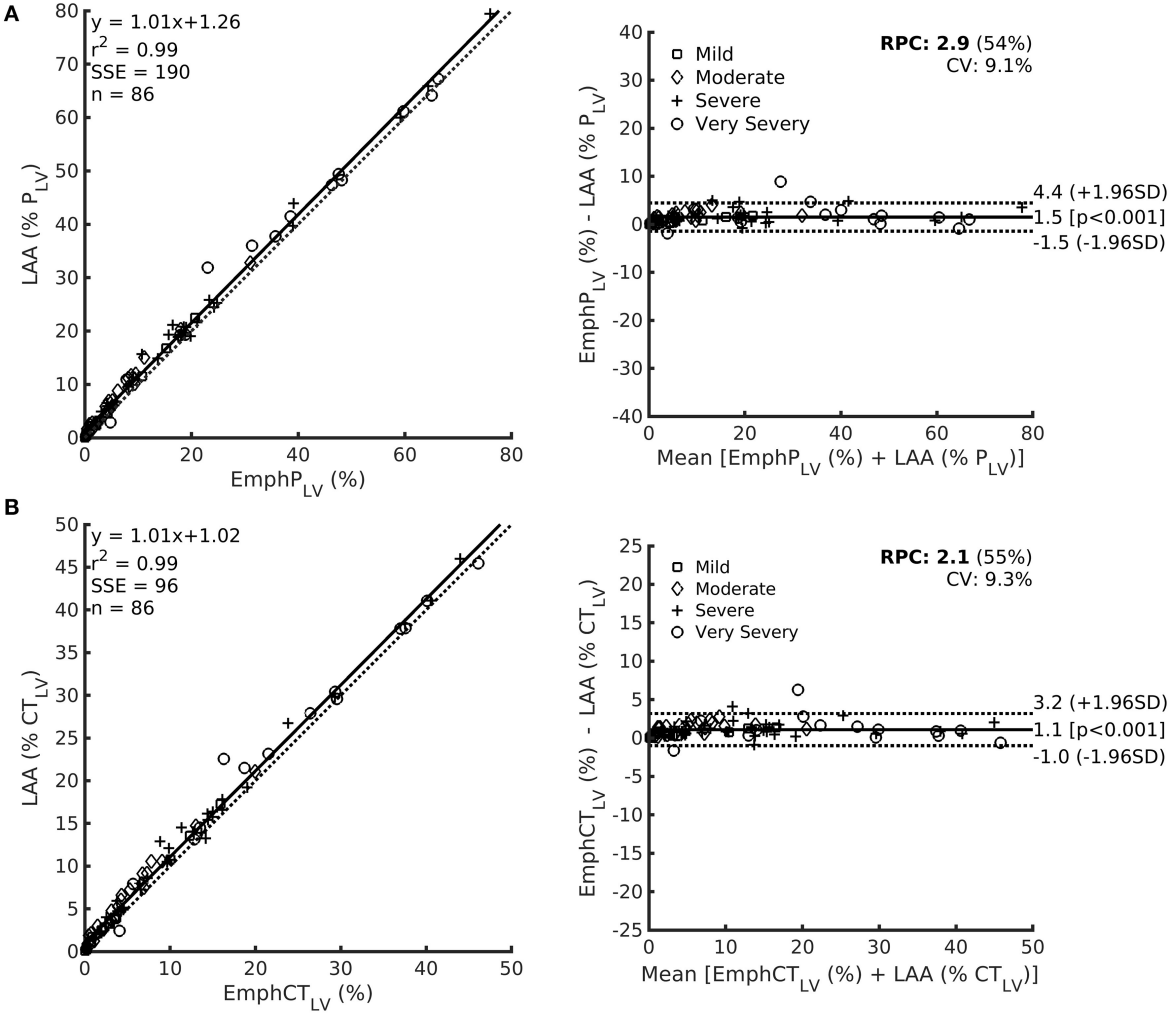
Left: Relationship among LAA expressed as the predicted lung volume LAA (%P_LV_) in row **(A)** or CT lung volume LAA (%CT_LV_) in row **(B)** and EmphP_LV_ (upper) or EmphCT_LV_ (lower) with each respective Bland–Altman bias plot (right). Noteworthy, even though a significant bias is observed, the magnitude of such a bias is always lower than 5%.

A very strong correlation was observed between LAA(%P_LV_) and EmphP_LV_ (Figure 6A) as well as between LAA(%CT_LV_) and EmphCT_LV_ (Figure 6B). Although the LAA estimations were always significantly higher than EmphCT_LV_ or EmphP_LV_ (Table 1), the bias ranged ~1.1–1.5% of the measured or predicted lung volume (Figure 6).

The Spearman's correlation coefficients between EmphP_LV_, EmphCT_LV_, LAA (%P_LV_), or LAA (%CT_LV_) and RV, TLC, FEV_1_, DL_CO_, DL_CO_/VA, and FVC are provided in Table 2. In general, all the CT-derived indices moderately to strongly correlated with RV, TLC, FEV_1_, and DL_CO_ and very weakly with FVC (Table 2).

**Table 2:** Spearman's correlation coefficients between QUALI-derived emphysema indices (EmphP_LV_ and EmphCT_LV_), LAA index obtained directed from CT densitometry and pulmonary function test variables.

	**Pulmonary function test variables**						
	**RV**	**TLC**	**FEV_**1**_**	**DL_**CO**_**	**DL_**CO**_/VA**	**FVC**	**RV/TLV**
EmphP_LV_	0.656	0.586	−0.603	−0.581	−0.594	−0.139	0.527
EmphCT_LV_	0.611	0.517	−0.602	−0.603	−0.606	−0.175	0.518
LAA (%P_LV_)	0.656	0.580	−0.598	−0.594	−0.610	−0.143	0.520
LAA (%)	0.602	0.508	−0.589	−0.617	−0.608	−0.175	0.514

*RV, Residual volume; TLC, Total lung capacity; FEV_1_, Forced expiratory volume measured during the first second; DLCO, Diffusing capacity for carbon monoxide; VA, Alveolar volume; LAA (%P_LV_), Low attenuation areas expressed as a percentage of the predicted lung volume; LAA (%CT_LV_), Low attenuation areas expressed as a percentage of CT measured lung volume; EmphP_LV_(%), QUALI-derived emphysema volume expressed as a percentage of the predicted lung volume; EmphCT_LV_ (%), QUALI-derived emphysema volume expressed as a percentage of CT measured lung volume; Orange squares marked strong (r = 0.60–0.79), yellow moderate (r = 0.40–0.59), and gray very weak (r = 0.00–0.19) Spearman's correlations*.

**Figure 7:**
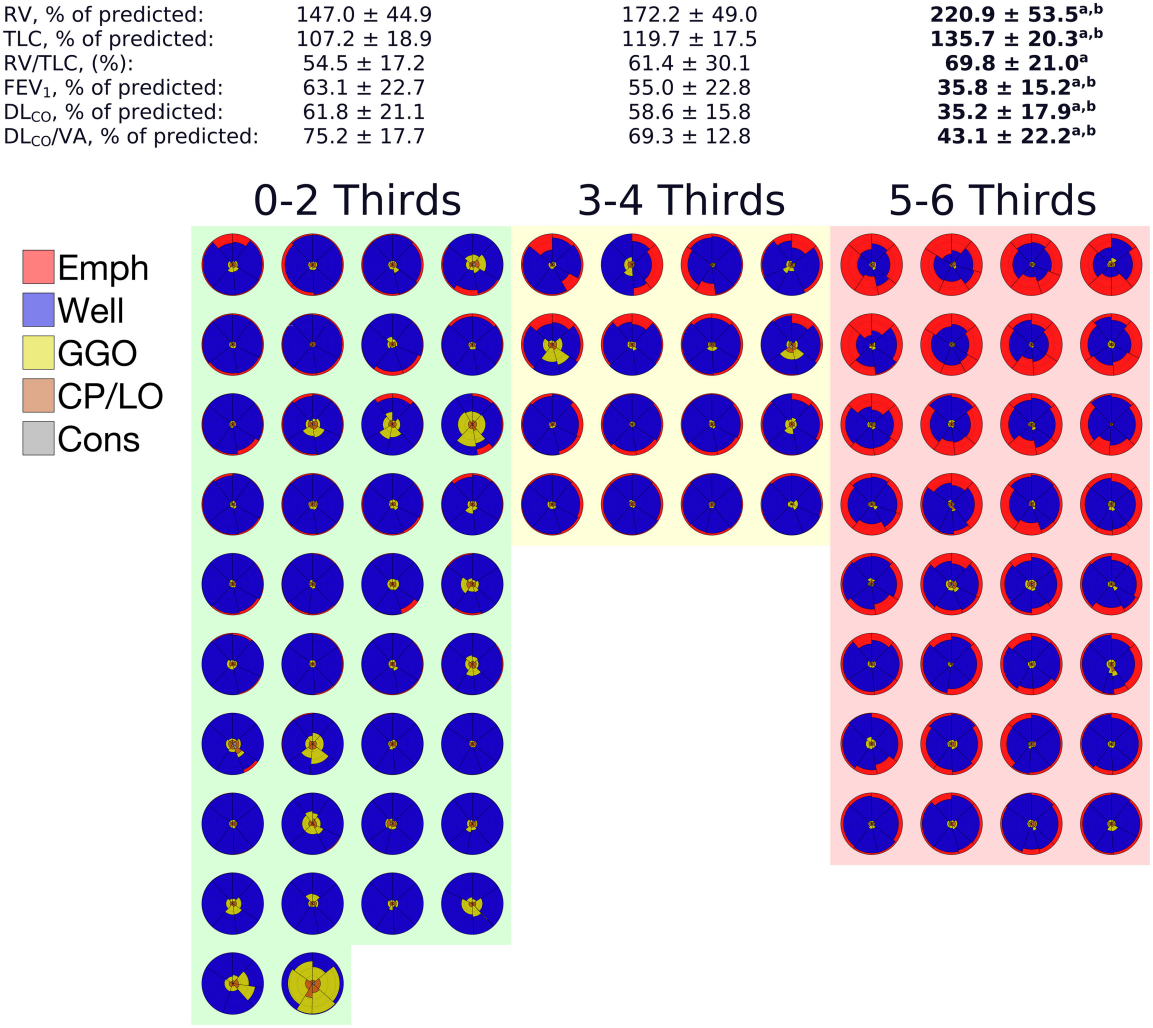
Means and SDs of the pulmonary function tests (PFTs) data grouped according to the number of segmented thirds affected with at least 5% of emphysema adjusted to the predicted lung volume. The glyphs pertaining to each patient in a given group are also depicted. In each glyph, emphysema is colored in red, GGO in yellow, CP/LO in orange, and consolidation in gray. Note that a significant impairment of PFTs variables was observed only when 5 or 6 lung thirds were emphysematous, notwithstanding the emphysema extent. ^a^Indicates a significant difference from 0 to 2 thirds affected by emphysema and a *p* < 0.001. ^b^Indicates a significant difference from 3 to 4 thirds affected by emphysema and a *p* < 0.05.

After dividing the left and right lungs in 3 thirds, EmphP_LV_ was assessed for each segmented lung region. Thus, when emphysema was present in more than 4 lung thirds, the patients presented a significantly higher RV, TLC, and RV/TLC ratio and a significantly lower FEV_1_, DL_CO_, DL_CO_/VA than the patients with 1–4 emphysematous segmented regions of the lungs (Figure 7).

## Discussion

Recently, we reported the performance of a software (QUALI) for automated classification and quantification of coronavirus disease-2019 (COVID-19) pneumonia extent from chest CT (19). In the present study, we included emphysema as another radiological pattern in QUALI and aimed to quantify emphysema extent and its regional distribution in the lungs. The updated QUALI system presented an excellent performance in classifying emphysema, with 99.4% agreement with consensual visually assigned emphysema areas by the two independent radiologists (Figure 3).

Even though the emphysematous changes recognized on density-based CT quantified emphysema (21, 30) have been proven to present strong associations with airflow obstruction, worsening the GOLD severity and air trapping (31, 32), the extent of pulmonary emphysema is usually calculated by considering the volume of areas identified as being emphysematous divided by the TLV measured by CT.

However, hyperinflation is a major characteristic of COPD patients with emphysema and correlates with disease severity (16). Thus, worsening of emphysema might imply an increased TLV leading to a progressive underestimation of emphysema extent by CT-derived indices. This fact could be particularly important in the follow-up studies to assess the rate of emphysema progression, and several approaches have been advanced to overcome such limitation (18, 33, 34).

Hence, we proposed to express all CT-derived emphysema indices as a percentage of the predicted TLV using a mathematical model that estimates the TLV of healthy subjects under identical conditions to those existing during the acquisition of chest-CT images (23).

In fact, all CT-derived emphysema estimates calculated both from an ANN-based classifier (EmphyCT) or from densitometric CT-derived analysis (LAA) consistently tend to underestimate the amount of emphysema when compared with the same indices adjusted to the predicted lung volume (Table 1 and Figure 5). Although the correlations between the emphysema indices adjusted by the predicted lung volume and pulmonary function did not differ from those adjusted by measured lung volume, the present data suggest that importance of recording the height of patient in the DICOM header file as a routine procedure in radiology department to calculate the predicted lung volume and avoid the underestimation of emphysema severity.

In addition, we should be aware that PFTs are conventionally performed with seated individuals, volumes are higher in the sitting position (35) and that mild and locally restricted emphysema can be identified on CT, but these radiologic changes may not affect the pulmonary function (5, 36).

Thus, we can probably highlight that the CT-derived emphysema indices should be adjusted by the predicted lung volume to better estimate emphysema extent especially in the patients with more severe GOLD emphysema severity classification. Future studies must also evaluate the influence of such a strategy on the evaluation of the progression of emphysema. Hence, we emphasize the importance of collecting the height of the patient and inserting it in the DICOM header file as a routine procedure in the radiology departments.

A very strong correlation between LAA and QUALI-derived emphysema indices (Figure 6) is quite expected as both use density characteristics in their computation. In LAA index, all voxels with densities equal to or below −950 HU are automatically assigned as emphysema areas. In fact, this strategy seems to overestimate the emphysema extent by the inclusion of low density but still, normal parenchyma voxels due to some density overlap between highly inflated normal areas and emphysema regions (Figure 4). Several studies also report the dependence of image acquisition and reconstruction parameters as potential limitations for lung densitometry analysis (17, 18, 33, 34, 37). This drawback might also affect the QUALI-derived emphysema indices. However, we emphasize that the same convolution sharp kernel filter was used in all the calculations, as recommended (37).

A potential advantage of the QUALI-derived emphysema indices compared to LAA densitometry method lies in its unique capability of identifying other parenchymal radiological patterns in addition to emphysema, since the overlap between COPD and other lung parenchymal diseases is common (30, 38, 39). Moreover, more complex computational methods using convolutional neural networks (30) that account not only to voxel density but also for voxel texture can be easily incorporated into QUALI soon.

Regarding emphysema distribution, our data showed that not just total emphysema extent but also a more homogenous emphysema distribution occurring in almost all lung regions seems to be present in patients with worse airflow obstruction, diffusing capacity, and hyperinflation (Figure 7). However, this finding must be cautiously interpreted because several studies report that the occurrence of emphysema in the middle and lower lung thirds seems to be the main contributor to lung function decline (40–42). Conversely, it has also been reported that independently of the emphysema extent, heavy (former) smokers with upper lobe-predominant CT-quantified emphysema exhibit a faster decrease in lung function than those with lower lobe-predominant CT-quantified emphysema (4).

The complexity between the emphysema progression and the compensatory pulmonary mechanisms to optimize gas-exchange might explain the relevance of regional distribution of emphysematous areas and its impact on the pulmonary. In fact, both emphysema extent and the regional occurrence of emphysema take place in the face of disease progression, thus rendering the isolation of their individual contribution or even to determine which one prevails was quite difficult (13, 41).

The present study presents some limitations. First, the female gender is not well-represented, but this lower proportion reflects daily clinical practice. Second, the disparity between scanners, scanner models, and image reconstruction kernels limit the application of both LAA and QUALI derived indices application in the clinical scenario. As a mitigation procedure, we used the same convolution kernel (sharp) and with the same slice thickness in all the studied images as previously recommended (37). Third, at the beginning of our study, we excluded 28 patients because the segmentation tool was not capable of performing segmentation due to their very hyperinflated lungs. This is an undesirable limitation since it prevents the use of our ANN in patients that would putatively benefit from it to a greater extent. Additionally, the absence of these patients could cause an overestimation of the correlation between lung function and the extent of emphysema as a percentage of the CT_LV_. Additionally, the division of the right and left lung into thirds represents only an approximation of the actual anatomy of the lung lobes. Certainly, segmentation into lobes considering each respective fissure would be very laborious and would require more computational resources.

To overcome such problems, we are working to apply a more complex convolutional neural network that does not need previous segmentation of the CT scans and works independently of the image reconstruction kernels used and will be able to automatically segment all the pulmonary lobes.

Apart from these limitations, the CT-derived emphysema indices can be adjusted to the predicted lung volume, being a simple and fast estimate of emphysema extent that can be obtained with minimal user intervention. Moreover, since COPD patients with emphysema benefit from long-term follow-up (29, 43), these are considerable advantages over visual evaluations of CT scans that are time-consuming, encompass interobserver variability, and are unable to quantify emphysema (44). Certainly, CTpLV is a potential index for the proper assessment of emphysematous lungs, however, the significance of CTpLV in clinical and research fields should be better elucidated in future studies.

In summary, in this group of COPD patients with pulmonary emphysema, the automatic quantification of the CT-derived emphysema indices adjusted to the predicted TLV enabled the quantification of emphysema extension and its regional distribution. The latter presented a moderate to strong correlation with pulmonary function tests parameters, especially with those associated with air trapping and hyperinflation. Pulmonary function impairment seemed to be more important in the patients with higher emphysema volumes and with a more homogeneous emphysema distribution among upper, middle, and lower lungs.

## Data Availability Statement

The raw data supporting the conclusions of this article will be made available by the authors, without undue reservation.

## Ethics Statement

The paper was submitted to the Centro Hospitalar de São João (CHSJ) Research Ethics Committee, who approved the present study (CES 56/21), and that it complied with the current national and international standards. As this is a retrospective study without intervention the consent form was waived by the respective ethics committees.

## Author Contributions

PG: capture and organization of clinical data, image processing and segmentation, analysis of results and statistical evaluation, and writing of the text and submission of the article. HB: capture and organization of clinical data, results discussion, and draft review. AC: capture of clinical data. AL: capture and organization of clinical data. AG: image processing and analysis of results and theoretical development of the neural network. RR and WZ: results discussion and draft review. ASC: image processing and analysis of results, theoretical development of the neural network and the computation method of voxel-to-voxel analysis, results discussion, and draft review. All authors approved the final version of the manuscript.

## Funding

This research was supported by the Brazilian Council for Scientific and Technological Development (Conselho Nacional de Desenvolvimento Científico e Tecnológico, Grants Nos. 302702/2017-2 and 302839/2017-8) and the Rio de Janeiro State Research Supporting Foundation (Fundação de Amparo à Pesquisa do Estado do Rio de Janeiro, Grants Nos. E-26/211.867/2016, E-26/202.785/2017, E-26/203.001/2018), and by national funds through FCT, Cardiovascular R&D Center – UnIC (UIDB/00051/2020 and UIDP/00051/2020).

## Conflict of Interest

The authors declare that the research was conducted in the absence of any commercial or financial relationships that could be construed as a potential conflict of interest.

## Publisher's Note

All claims expressed in this article are solely those of the authors and do not necessarily represent those of their affiliated organizations, or those of the publisher, the editors and the reviewers. Any product that may be evaluated in this article, or claim that may be made by its manufacturer, is not guaranteed or endorsed by the publisher.
